# The Properties of Alpha Risk Parity Portfolios

**DOI:** 10.3390/e24111631

**Published:** 2022-11-10

**Authors:** Jérôme Gava, Julien Turc

**Affiliations:** 1 Department of Economics, Ecole Polytechnique, Institut Polytechnique de Paris, 91120 Palaiseau, France; 2 BNP Paribas, 75009 Paris, France

**Keywords:** portfolio optimization, risk parity, risk budgeting, generalized entropy, statistical divergence, information theory, portfolio selection, investment risk, diversification

## Abstract

Risk parity is an approach to investing that aims to balance risk evenly across assets within a given universe. The aim of this study is to unify the most commonly-used approaches to risk parity within a single framework. Links between these approaches have been identified in the published literature. A key point in risk parity is being able to identify and control the contribution of each asset to the risk of the portfolio. With alpha risk parity, risk contributions are given by a closed-form formula. There is a form of antisymmetry—or self-duality—in alpha risk portfolios that lie between risk budgeting and minimum-risk portfolios. Techniques from information geometry play a key role in establishing these properties.

## 1. Introduction

Risk parity is an approach to investing that aims to balance risk evenly across a diversified range of assets. Risk parity portfolios have been shown to be more diversified than traditional 60/40 portfolios [1]. Risk parity is a sensible choice for investors who know which assets to buy but do not wish to express views on expected returns across these assets. This approach can also be applied to baskets of systematic investment strategies [2].

Risk parity can be defined in various ways. In their simplest form, risk parity portfolios weight investments according to an estimate of their volatility. A more advanced approach considers the contribution of each asset to the risk of the portfolio [3]. Links have been established between three important approaches. Equal weights and minimum variance are limit cases of a constrained risk-minimization problem that includes risk budgeting [3]. Starting with equal weights and iteratively applying risk budgeting leads to minimum variance [4]. Conversely, starting with minimum variance and applying covariance shrinkage leads to equal weights [5,6,7,8].

This study investigates these links using techniques from information theory and geometry. Using entropy as a measure of portfolio diversity was proposed in [9] and has been subject to further research. The entropy of portfolio returns has also been considered as a possible risk measure [10]. The two approaches differ. One considers the distribution of weights across assets, the other is based on the statistical distribution of financial returns.

Alpha risk parity is a generalization of the equal risk contribution approach of Maillard, Teiletche, and Roncalli [3]. A generalized log-barrier based on Tsallis’ *q*-logarithm [7] ensures a minimum level of diversity in the allocation. The contribution of each asset to the risk of the portfolio is given by a closed-form formula. A key parameter that is related to Tsallis’s entropic index determines how much weights can diverge from risk budgets. Adjusting the parameters leads to equal weights or minimum variance as the two ends of the spectrum, and to risk budgeting as a compromise.

Alpha risk parity is applied to a selection of major equity, bond, and commodity futures. Equal-weighted portfolios are the least invariant to duplication [11] and the most diversified across assets. Minimum-variance portfolios are at the other end of the spectrum. Equal risk-contribution portfolios are a compromise between these two extremes; however, they are much closer to equal weights than they are to minimum risk. Increasing the alpha parameter is a sensible way to loosen the diversity constraint and give a greater role to risk minimization.

## 2. Theoretical Framework

### 2.1. Literature Review

#### 2.1.1. Risk Parity

Naive risk parity consists of scaling individual assets by their estimated risk. Why this approach makes sense has been a subject of debate. Risk parity portfolios may benefit from investors’ aversion to financial leverage [12], although the cost of leverage and rebalancing may offset these benefits [13]. Although most studies on risk parity focus on allocation across asset classes, the approach may be applied to single shares [14], equity sectors [15], and corporate bonds [16]. Equal risk contributions extends naive risk parity to the correlated case [3]. This approach can be extended further to any risk budgets [17].

Minimizing risk under constrained exposure is another way to allocate a portfolio without expressing views on future returns [18,19]. When assets are uncorrelated, minimum variance is achieved by scaling individual assets by their variance. Some authors [20] use the term of ‘risk-based portfolios’ for any approach that relies solely on risk. Naive risk parity, equal risk contributions, minimum variance, and maximum diversification portfolios [11] are risk-based portfolios.

Risk criteria are usually selected from two main classes [21]. Volatility and downside volatility are deviation measures [22]. Conditional value-at-risk [23] and the power-spectrum measure [2] are spectral risk measures [24] that can be applied to risk parity portfolios after proper centering. See [2] for a review of tail-risk measures and their application to risk parity portfolios with and without dependencies.

There is no precise definition of risk parity in the general case. In the literature on hierarchical allocation, risk parity across clusters is achieved by using equal risk contributions [25,26], or minimum variance [27]. In [25], portfolios are allocated across clusters using equal weights. Following [3,4], this study proposes a unified framework for these approaches.

Some approaches to risk parity are dramatically impacted by misspecification in the investment universe. Duplication invariance is introduced in [11] and discussed in [4]. A construction process is invariant to duplication if portfolios remain the same when an asset is listed twice in the investment universe. Minimum variance are duplication-invariant and highly concentrated. Equal-weighted portfolios are sensitive to duplication, although they consider correlations between assets. Measures of concentration and diversity are reviewed in [20].

#### 2.1.2. Using Entropy and Information Geometry for Portfolio Allocation

This study is related to the literature on using the entropy of the weights of a portfolio as a measure of diversification. Concentration measures, Shannon, Rényi and Tsallis entropy, and various divergence measures are reviewed in [20] in the context of portfolio allocation. Controlling the Tsallis entropy of a portfolio allocation is a way to consider missing information [9]. Shannon’s cross-entropy can be used in order to shrink an optimal portfolio towards a predetermined allocation [28]. It can be used for optimizing portfolios based on their expected return [29] or on higher-order moments [30].

Another strand of the literature applies entropies to the distribution of financial returns. The Shannon entropy of the returns of a portfolio may be considered as a risk measure [10]. Entropies of returns and weights may also be combined into a single optimization problem [31,32].

Optimal transport can be applied in order to measure the distance between the distribution of returns of a portfolio and a target distribution [33,34,35]. It is also possible to use classical, Euclidean distances for controlling the tracking error of a portfolio [36]; however, Euclidean distances do not account for the fact that weights of long-only portfolios cannot be negative.

### 2.2. Notations and Assumptions

In this study, portfolios are long-only. They are represented by *n*-dimensional vectors of positive weights $x=(x_{1},\ldots ,x_{n})$. x≥0 means that $x_{i}\geq 0$ for all assets *i*. The sum of weights of a portfolio is called exposure. A fully-invested portfolio has an exposure of 100% without financial leverage. In this case, *x* is an element of the (n−1)-dimensional probability simplex $\Delta_{n-1}=\left\{\left(x_{1},\ldots ,x_{n}\right)\;\left|\;x\geq 0\;\mathrm{and}\;\sum_{i=1}^{n}x_{i}=1\right.\right\}$. In this paper, $\Delta_{n-1}$ is called ‘the simplex’. The open probability simplex—or open simplex—is the topological interior of the simplex. ${\overset{˚}{\Delta }}_{n-1}=\left\{\left(x_{1},\ldots ,x_{n}\right)\;\left|\;for\;all\;i,\;x_{i}>0\;\mathrm{and}\;\sum_{i=1}^{n}x_{i}=1\right.\right\}$.

A long-only portfolio that is exposed to all assets with unconstrained exposure belongs to the set $R_{+}^{*n}=\left\{\left(y_{1},\ldots ,y_{n}\right)\;\left|\;y_{i}>0\right.\right\}$ of positive measures. Normalizing portfolio y≠0 means defining a new portfolio $\tilde{y}$ within the simplex as ${\tilde{y}}_{i}=\frac{y_{i}}{\sum_{j=1}^{n}y_{j}}$ for all 1≤i≤n. Assets with positive weights are the support of the portfolio.

Risk budgets *b* are elements of the simplex. Weights $b_{i}$ are supposed to be positive. The case when $b_{i}=0$ is discussed in Section 2.7 and Section 4.2. A divergence from portfolio *x* to portfolio *b* is noted $D\left(b\|x\right)$.

*R* is any risk measure that is differentiable on $R_{+}^{*n}$, (positively) homogeneous of degree 1 and convex. The homogeneity condition means that risk must be proportional to portfolio exposure. R(w) is assumed to be positive if w≠0. Deviation risk measures [22] are good candidates if they verify the differentiability criterion. Volatility is a deviation measure. Other risk measures, such as the conditional value-at-risk, can be used provided they are adjusted in such a way that R(0)=0.

For any $x\in \Delta_{n-1}$ and positive numbers $k_{i}$, ‘$x_{i}\propto k_{i}$’ means that for all 1≤i≤n, $x_{i}=\frac{k_{i}}{\sum_{j=1}^{n}k_{j}}$.

### 2.3. Equal Risk Contributions and Risk Budgeting

This section is a more detailed review of the literature about the risk-budgeting approach to risk parity.

#### 2.3.1. The Direct Approach

Risk budgeting aims to control the contribution of each asset to the risk of the portfolio. This approach is introduced in [17] and thoroughly investigated in [4]. For any allocation *y*, let R(y) be the risk of the corresponding portfolio. Euler’s decomposition applied to the homogeneous risk criterion implies that $R\left(y\right)=\sum_{i=1}^{n}RC_{i}\left(y\right)$, where risk contribution $RC_{i}$ is defined as:

(1) $$RC_{i}\left(y\right)=y_{i}\partial_{i}R\left(y\right).$$

Risk contributions are proportional to the exposure of the portfolio.

In equal risk contribution portfolios, the contributions equal each other. This approach can be extended to any percentage breakdown $\left(b_{1},\ldots ,b_{n}\right)$ of risk. For any weights *b* from the open simplex, the risk-budgeting portfolio is defined for each asset *i* by:

(2) $$\frac{RC_{i}}{R}\left(y\right)=b_{i}.$$

The weights $b_{i}$ are the risk budgets. The risk-budgeting portfolio is the portfolio of exposure 1 that solves Equation (2). *b* is called the budget-weighted portfolio. When the budgets equal each other, *b* is the equal-weighted portfolio.

In contrast, the risk contributions in minimum-risk portfolios are determined by the weights of the portfolio rather than by any risk budgets.

(3) $$\mathrm{For}\;\mathrm{all}\;i,\;\frac{RC_{i}}{R}\left(y\right)=y_{i}.$$

Equation (3) is true even when the portfolio is long-only (see [4] or Appendix A). In this study, minimum-risk portfolios are always long-only. There may be multiple minimum-risk portfolios, unless risk measure *R* is strictly convex on the simplex. This property is verified if volatility is the risk measure and if no assets are 100%-correlated.

Risk-budgeting portfolios can be found by numerically minimizing any differences between risk contributions and risk budgets under constrained exposure [37]. They can also can be found by solving a variational problem.

#### 2.3.2. The Variational Approach

As shown in [3,4], the solution $y^{*}$ to the following optimization problem also solves the risk-budgeting Equation (2):

(4) $$\min\left\{R\left(y\right)|\;y\geq 0,\;-\sum_{b_{i}>0}b_{i}\log\left(\frac{y_{i}}{b_{i}}\right)\leq c\right\}.$$

This surprising property is established by minimizing the Lagrangian function:

(5) $$L\left(y,\mu \right)=R\left(y\right)-\mu \sum_{i=1}^{n}b_{i}\log\left(y_{i}\right).$$

As portfolios in problem (4) have an unconstrained exposure, optimal portfolio $y^{*}$ must be normalized. The resulting portfolio $\tilde{y}$ also solves the risk-budgeting equation. Constraint *c* can take any value, and all such values lead to the same normalized portfolio $\tilde{y}$ (see Appendix G for a proof in a more general setting).

There is an intuitive side to variational problem (4). The nonlinear constraint involves a log-barrier function which increases to infinity when weights are too small [38]. The variational approach to risk budgeting minimizes risk under a constraint that makes it impossible to leave any asset unallocated.

Starting with given risk budgets and iteratively applying the risk-budgeting procedure leads to the minimum-risk portfolio after an infinite number of iterations [4]. When volatility is the risk measure, shrinking the covariance matrix forces equal risk contribution portfolios towards equal weights [5]. The impact of shrinkage in risk parity portfolios and other portfolio construction processes is reviewed in [39].

If volatility is the risk measure and assets are uncorrelated, the equal risk contribution portfolio is found by weighting each asset according to its volatility and normalizing the resulting portfolio. This rule also holds when correlation is constant across all pairs of assets (see [4] or Appendix D). With general risk budgets, risk-budgeting portfolios that involve uncorrelated assets are given by [17]:

(6) $$y_{i}^{*}\propto \frac{\sqrt{b_{i}}}{\sigma_{i}}.$$

This formula does not extend to correlated assets unless all risk budgets are equal.

### 2.4. A Generalized Approach to Risk Budgeting

#### 2.4.1. Using a Generalized Logarithm

Alpha risk parity is based on replacing the logarithm in program (4) with the *q*-logarithm, which was introduced by Tsallis in [7]. For any entropic index q≥0 and any y>0, the *q*-logarithm is defined by $\log_{q}\left(y\right)=\log\left(y\right)$ if q=1 and by

(7) $$\log_{q}\left(y\right)=\frac{1}{1-q}\left(y^{1-q}-1\right)$$

if q≠1. If q<0 and x=0, this definition can be extended to a finite value. If q=0, the *q*-logarithm is defined for any value of *y*. For any other values of *q* and *y*, the *q*-logarithm is −∞. The same convention is applied to the natural logarithm.

In this study, the *q*-logarithm is parametrized by

(8) $$q\left(\alpha \right)=\frac{1-\alpha }{2}\;\mathrm{for}\;\mathrm{any}\;\alpha \leq 1.$$

The reason for this change of parameter is related to self-duality and compatibility to Amari’s divergences, as discussed in Proposition 5 and in Section 2.6.3.

**Figure 1:**
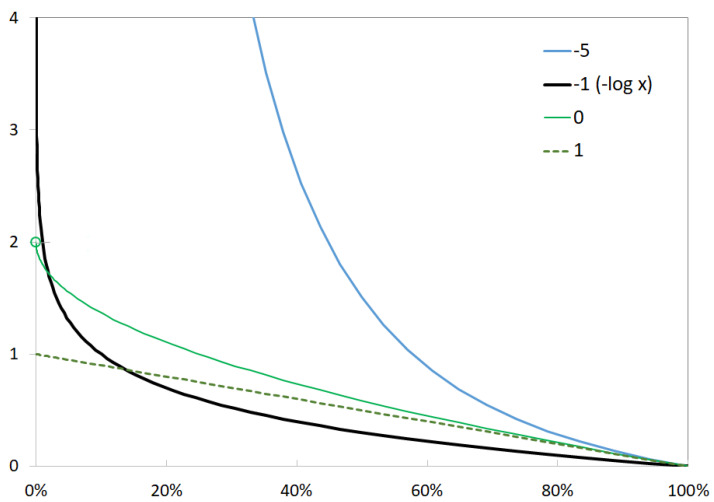
The negative *q*-logarithm for various values of α.

The negative *q*-log is always strictly decreasing. It is a closed and strictly convex function if q>0 or equivalently if α<1. As shown in Figure 1, the negative *q*-logarithm can be used as a log-barrier. With the parametrization in α, the function takes a finite value when −1<α≤1. It is affine when α=1.

The natural logarithm maps products into sums. This morphism property explains why risk-budgeting portfolios do not depend on the exact value of constraint *c* in problem (4). The *q*-logarithm satisfies a quasimorphism property that is detailed in Appendix B. The inverse of the *q*-logarithm is the *q*-exponential function $\exp_{q}$.

#### 2.4.2. Introducing Alpha Risk Parity

**Definition** **1.** *For any α≤1 and associated entropic index $q=q\left(\alpha \right)$, for any constraint c such that*

(9) $$c<\frac{2}{1+\alpha }\;\mathrm{if}\;-1<\alpha \leq 1,$$

alpha risk parity is achieved by normalizing solutions $y_{q}^{*}\left(c\right)$ to

(10) $$\min\left\{R\left(y\right)|\;y\geq 0,\;-\sum_{b_{i}>0}b_{i}\log_{q}\left(\frac{y_{i}}{b_{i}}\right)\leq c\right\}.$$

Alpha risk parity portfolios are given by $\tilde{y}\left(c\right)=y_{q}^{*}\left(c\right)/\sum_{i}{\left(y_{q}^{*}\left(c\right)\right)}_{i}$. The set of admissible portfolios is denoted by $S_{q}\left(c\right)$.

Strict convexity ensures uniqueness of $y_{q}^{*}\left(c\right)$ and the alpha risk parity portfolio when α<1. If α=1, alpha risk parity leads to the minimum-risk approach. Risk budgeting is a particular case for α=−1.

In this definition, condition (9) guarantees that the generalized constraint in problem (10) is active. If this condition is not satisfied, the null portfolio is a solution to the optimization problem.

Constraint *c* can be any negative number, because the negative *q*-logarithm takes arbitrarily large negative values for large weights. As noted in Appendix C, the properties of the *q*-logarithm at 0 ensure that solutions to problem (10) have positive weights. From a theoretical standpoint, constraints $y_{i}\geq 0$ may be taken out of the problem. However, these constraints can play a helpful role in numerical optimization.

For any α≤1 such that α≠−1, the alpha log-barrier is explicitly given for all $y\in R_{+}^{*n}$ by:

(11) $$F_{\alpha }\left(b,y\right)\;=\;-\sum_{b_{i}>0}b_{i}\log_{q(\alpha )}\frac{y_{i}}{b_{i}}\;=\;\frac{2}{1+\alpha }\left(1-\sum_{b_{i}>0}b_{i}^{\frac{1-\alpha }{2}}y_{i}^{\frac{1+\alpha }{2}}\right).$$

$F_{\alpha }$ can be extended by continuity on $R_{+}^{n}$ respective to *y* if α>−1.

### 2.5. Properties of Alpha Risk Parity

#### 2.5.1. A Simple Case

**Proposition** **1.** *Let $\sigma_{i}$ be the volatility of asset i. When volatility is the risk measure and assets are uncorrelated, the alpha risk parity portfolio is*

(12) $${\tilde{y}}_{i}\;\propto \;\frac{b_{i}^{\frac{1-\alpha }{3-\alpha }}}{\sigma_{i}^{\frac{4}{3-\alpha }}}.$$

This equation is established in Appendix D.

Using α=1 leads to the inverse-variance portfolio, which is also the minimum-variance portfolio. Risk budgeting corresponds to the case α=−1, and Equation (12) implies Equation (6). If α→−∞, alpha risk parity converges to budget weighting *b*.

With equal risk budgets, the alpha risk parity portfolio is determined by

(13) $${\tilde{y}}_{i}\;\propto \;\frac{1}{\sigma_{i}^{\frac{4}{3-\alpha }}}.$$

Naive risk parity, which is achieved by taking α=−1, amounts to volatility scaling. As observed in [3], equal risk contribution is a compromise between equal weights and minimum variance. Alpha risk parity, which scales assets by a power of their volatility, connects the three portfolios by moving α from −∞ to 1 in Equation (13).

With equal risk budgets, alpha risk parity leads to equal weights if correlation and volatility are constant. When α=−1, the optimal portfolio is given by Equation (6) if the correlation is constant. Otherwise, no simple solution emerges—as noted in [4,17] in the case of risk budgeting.

#### 2.5.2. Independence from the Constraint

As shown in Proposition A1, admissible sets $S_{q}\left(c\right)$ associated with various constraints *c* can be uniformly scaled into each other.

**Proposition** **2.** For any α≤1 and associated entropic index $q=q\left(\alpha \right)$, the optimal portfolios $y_{q}^{*}\left(c\right)$ are proportional to each other. If c verifies condition (9),

(14) $$y_{q}^{*}\left(c\right)=\exp_{q}\left(-c\right)\;y_{q}^{*}\left(0\right).$$

*In the particular case of α=1, there may be multiple solutions. Equation (14) establishes a mapping between the sets of solutions associated with constraints c and 0.*

After normalization, alpha risk parity portfolios $\tilde{y}$ do not depend on the exact value of constraint *c*.

**Proposition** **3.** *All constraints c that verify condition (9) lead to the same alpha risk parity portfolios.*

Proofs of Propositions 2 and 3 are detailed in Appendix E. Proposition (3) shows that any admissible value of *c* may be used for finding an alpha risk parity portfolio. In practical applications, using c=0 is a sensible choice. The resulting optimization problem is summed up in the following proposition.

**Proposition** **4.** *Here is an explicit formulation of the alpha risk parity problem using c=0:*

- *For any α<−1, the alpha risk parity portfolios are found by normalizing the solution to optimization problem:*
(15) $$\min\left\{R\left(y\right)|\;for\;all\;i,\;y_{i}\geq 0,\;and\;\sum_{i}b_{i}^{\frac{1-\alpha }{2}}y_{i}^{\frac{1+\alpha }{2}}\leq 1\right\}.$$
- *For α=−1, the alpha risk parity portfolio is the risk-budgeting portfolio. It is found by normalizing the solution to:*
(16) $$\min\left\{R\left(y\right)|\;for\;all\;i,\;y_{i}\geq 0,\;and\;\sum_{i=1}^{n}b_{i}\log\left(\frac{y_{i}}{b_{i}}\right)\geq 0\right\}.$$
- *For −1<α≤1, alpha risk parity portfolios are found by normalizing solutions to:*
(17) $$\min\left\{R\left(y\right)|\;for\;all\;i,\;y_{i}\geq 0,\;and\;\sum_{i}b_{i}^{\frac{1-\alpha }{2}}y_{i}^{\frac{1+\alpha }{2}}\geq 1\right\}.$$

The exposure of the nonnormalized portfolio verifies $\lambda^{*}>1$ for any value of α if $y^{*}\neq b$.

The last assertion in Proposition 4 is a consequence of the strict convexity of the alpha log.

**Proposition** **5.** *Alpha risk parity portfolios satisfy a self-duality property for −1<α<1. Within this range, alpha risk parity portfolios associated with parameter −α are solutions to*

(18) $$\min\left\{R\left(y\right)|\;y\geq 0,\;F_{\alpha }\left(y,b\right)\leq c\right\}.$$

*Changing the sign of α amounts to inverting the role of portfolios b and y in the optimization problem.*

Proposition 5 follows from Proposition 3 and from the fact that $F_{-\alpha }\left(y,b\right)=\frac{1+\alpha }{1-\alpha }\;F_{\alpha }\left(y,b\right)$ if α∉{−1,1} and b,y are within the open simplex.

#### 2.5.3. A Risk-Budgeting Equation for Alpha Risk Parity

The next proposition clarifies the link between risk budgets and reference weights $b_{i}$ and extends risk-budgeting Equation (2) beyond α=−1.

**Proposition** **6.** *The risk contribution of any asset i in an alpha risk parity portfolio is given by:*

(19) $$\frac{RC_{i}}{R}\left(\tilde{y}\right)\;\propto \;b_{i}\;{\left(\frac{{\tilde{y}}_{i}}{b_{i}}\right)}^{\frac{1+\alpha }{2}}.$$

*If α≠−1, Equation (19) can be written as:*

(20) $$for\;all\;i,\;{\tilde{y}}_{i}\;\propto \;b_{i}\;{\left(\frac{RC_{i}\left(\tilde{y}\right)}{b_{i}}\right)}^{\frac{2}{1+\alpha }}$$

The proof of this proposition is shown in Appendix F. It follows the same line of reasoning as the one presented in Section 2.3 for risk budgeting. Proposition 6 has a few interesting consequences:

- If α=1, Equation (19) $\Leftrightarrow RC_{i}/R={\tilde{y}}_{i}$. This is the risk-budgeting Equation (3) of minimum-risk portfolios.
- If α=−1, Equation (19) $\Leftrightarrow RC_{i}/R=b_{i}$. Alpha risk parity amounts to risk budgeting (see Equation (2)).
- If α→−∞, Equation (20) converges to $\tilde{y}\propto b_{i}$. Budget weighting is a limit case of alpha risk parity.

The weights $b_{i}$ play a double role in risk-budgeting Equation (19). They are risk budgets that partially control risk contributions. If α≠−1 and α<1, they also act as a reference portfolio because weights ${\tilde{y}}_{i}$ are compared with the $b_{i}$. This point is clarified in Section 2.6. Using α<−1 strengthens the role of *b* as a reference portfolio because any below-budget allocation leads to explosive risk contributions. Using α>−1 weakens the role of *b* as a reference portfolio and as risk budgets. By adjusting α, investors can determine how much they want to control portfolio weights and risk budgets.

Risk-budgeting Equation (19) may be used as a starting point to finding an alpha risk parity portfolio. However, the variational approach makes it possible to use numerical convex optimization and makes it easier to establish the uniqueness of the solution.

### 2.6. Utility Maximization under Divergence Constraint

#### 2.6.1. Risk Budgeting Portfolios and Maximum Utility

As noted in [40], ‘risk parity is not a traditional mean-variance objective function’. However, the risk parity—or risk-budgeting—problem is a form of utility maximization because the variational approach (4) minimizes a risk criterion. If this criterion is compatible with a utility function, then risk budgeting maximizes a utility function. Minimizing volatility amounts to maximizing a quadratic von Neumann–Morgenstern expected utility function with 0% expected returns. Minimizing a spectral risk measure amounts to maximizing an expected rank-based utility function [2,41].

Risk budgeting differs from traditional utility maximization because the portfolio in a variational problem (4) is optimized with unconstrained exposure. Optimizing with unconstrained exposure and normalizing afterwards implicitly assumes unlimited financial leverage. However, Refs. [3,4] show that equal risk contributions and risk budgeting solve a risk-minimization problem with constrained exposure.

Here is an outline of the proof. The portfolio $y^{*}$ that solves problem (4) is optimal among admissible portfolios of all exposures. It is also optimal among those whose exposure is equal to $\lambda^{*}=\sum_{i}y_{i}^{*}$. Using change of variable $y=\lambda^{*}x$, normalized portfolio $\tilde{y}$ solves the following problem among portfolios of exposure 1:

(21) $$\min\left\{R\left(x\right)|x\geq 0,\;\sum_{i=1}^{n}x_{i}=1,\;-\sum_{b_{i}>0}b_{i}\log\frac{x_{i}}{b_{i}}\leq d\right\}$$

In this problem, constraint *d* must be set to $d=c^{*}=c+\log\lambda^{*}$. Using c=0 leads to:

(22) $$c^{*}=\log\lambda^{*}.$$

In problem (21), the portfolio is optimized over the probability simplex. Therefore, using the logarithmic constraint amounts to controlling the Kullback–Leibler divergence $D_{KL}$ from portfolio *x* to portfolio *b*.

(23) $$\mathrm{For}\;\mathrm{all}\;b,x\in \Delta_{n-1},\;D_{KL}\left(b\|x\right)=-\sum_{b_{i}>0}b_{i}\log\frac{x_{i}}{b_{i}}.$$

In the theory of divergence functions, the Kullback–Leibler divergence is the *f*-divergence associated with the negative logarithm. Appendix H contains a brief summary on this account.

Formulation (21) is of little practical use because determining the divergence constraint requires prior knowledge of the exposure of the optimal portfolio. However, this formulation shows that risk budgeting is a rational approach to investing. When using the right risk criterion, risk budgeting maximizes a utility function with constrained exposure and constrained divergence to portfolio *b*. The risk budgets $b_{i}$ play the role of a reference portfolio, and divergence is constrained using a nonsymmetric statistical divergence function.

As noted in [3,17], the risk of optimal portfolio defined by (21) decreases with constraint *d*. It is maximal when d=0 and the portfolio is equal to the budget-weighted portfolio *b*. It is minimal when d=+∞. Risk budgeting is a compromise between these two extremes.

#### 2.6.2. Beyond Kullback–Leibler

The reasoning of Section 2.6.1 can be extended to alpha risk parity.

**Proposition** **7.** For any α≤1 and associated entropic index q, let $\lambda^{*}$ the exposure of optimal, nonnormalized portfolio $y_{q}^{*}\left(0\right)$ and

(24) $$c^{*}=-\log_{q}\left(\frac{1}{\lambda^{*}}\right).$$

*Alpha risk parity portfolios solve the following risk-minimization problem with constrained exposure:*

(25) $$\min\left\{R\left(x\right)|\;x\geq 0,\;\sum_{i=1}^{n}x_{i}=1,\;-\sum_{b_{i}>0}b_{i}\log_{q}\left(\frac{x_{i}}{b_{i}}\right)\leq c^{*}\right\}.$$

Proofs of these propositions are detailed in Appendix G.

Using the *q*-logarithmic constraint in problem (25) amounts to controlling the *q*-divergence $D_{q}$ from portfolio *x* to portfolio *b*.

(26) $$\mathrm{For}\;\mathrm{all}\;b,x\in \Delta_{n-1},\;D_{q}\left(b\|x\right)=-\sum_{b_{i}>0}b_{i}\log_{q}\frac{x_{i}}{b_{i}}.$$

The *q*-divergence [42], which is also known as quantum *q*-divergence [43], is the *f*-divergence associated with $f=-\log_{q}$ for q>0. For q=0, the *q*-divergence is formally defined as $1-\sum_{i}x_{i}$. It is not a divergence function due to a lack of strict convexity.

The *q*-divergence associated with α=0—or $q=\frac{1}{2}$—is symmetric. For $b,x\in \Delta_{n-1}$, it is related to the Hellinger distance *h* (see Equation (A14)) by

(27) $$D_{q(0)}\left(b\|x\right)=2\;h{\left(b,x\right)}^{2}.$$

When using the right risk criterion, alpha risk parity maximizes a utility function with constrained exposure. If α<1, deviations respective to portfolio *b* are controlled using a statistical divergence. In this case, the risk budgets $b_{i}$ play the role of a reference portfolio.

#### 2.6.3. Alpha Risk Parity and Alpha Divergences

For α<1, multiplying both sides of Equation (26) by $\frac{2}{1-\alpha }$ shows that alpha risk parity is constrained according to

(28) $$D_{\alpha }\left(b\|x\right)=\sum_{b_{i}>0}b_{i}f_{\alpha }\left(\frac{x_{i}}{b_{i}}\right).$$

$f_{\alpha }$ is an extension of the negative logarithm that was introduced by Amari [8]. If α=−1, $f_{\alpha }=-\log$. If α∉{−1,1}, for all x>0,

(29) $$f_{\alpha }\left(x\right)=\frac{4}{1-\alpha^{2}}\left(1-x^{\frac{1+\alpha }{2}}\right).$$

For any value of α, $f_{\alpha }$ is a strictly convex function. $D_{\alpha }$ is the *f*-divergence associated with $f_{\alpha }$. It is known as Amari’s alpha divergence.

Therefore, alpha risk parity also minimizes risk under constrained alpha divergence. Why this matters is twofold. Alpha divergences are the only flat divergences defined on the set of positive measures (see Appendix H). In comparison, the Kullback–Leibler divergence and its dual are the only flat divergences defined on the simplex. Alpha risk parity, which is based on the alpha divergence, is a natural extension of risk budgeting.

Furthermore, alpha divergences are also defined for α>1. In contrast, *q*-divergences are only defined for α≤1 in the α-parametrization. In the particular case of alpha risk parity, using $f_{\alpha }$ functions instead of the negative *q*-logarithm does not bring much because $f_{\alpha }$ is a strictly increasing function when α>1. In this case, the null portfolio is the only solution to problem $\min\left\{R\left(y\right)|y\geq 0,\;\sum_{i}b_{i}f_{\alpha }\left(\frac{y_{i}}{b_{i}}\right)\leq c\right\}$.

However, alpha divergences can be useful tools for controlling the allocation of more general maximum-utility portfolios. Some authors have proposed penalizing portfolios by the sum of squared weights [9,31]. This constraint does not belong to the family of *q*-divergences. However, taking α=3 and using equal risk budgets leads to $D_{\alpha }\left(b\|x\right)$$=\frac{1}{4}\left(n\;\sum_{i}x_{i}^{2}-1\right)$. Therefore, Amari’s alpha divergences can be used in order to extend the approach proposed in [9] to more general reference portfolios. The cross-entropy used in [28] can also be extended using an alpha divergence.

### 2.7. What Happens if Some Risk Budgets Are Equal to Zero?

It may be interesting to consider an investment universe that goes beyond the list of assets with a positive risk budget [4]. This problem can be approached by allocating a risk budget $b_{i}=0$ to some—but not all—assets in the universe. Divergences and log-barriers are based on assets with a positive risk budget. The variational approach to alpha risk parity combines one portfolio that is constrained by an alpha log-barrier with another that is managed similar to a minimum-risk portfolio. A common risk criterion is applied to the two portfolios.

Due to the logarithmic barrier, the allocation to assets with a positive risk budget cannot be too small. Assets with no risk budget are essentially allocated without any constraint. They enter the portfolio only if they contribute to a decrease in risk.

In terms of risk-budgeting formulas, assets with a positive risk budget verify the equation of alpha risk parity portfolio (19). Other assets verify minimum-variance Equation (3). Risk budgets must be summed over all assets.

## 3. Materials and Methods

The alpha risk parity algorithm is applied to a dataset that starts in January 1994 and ends on 10 August 2022. At each trading date, a risk-budget portfolio *b* is constructed by applying equal weights to the multi-asset reference universe shown in Table 1.

The indices represent the return received or paid when rolling financial futures. All returns are excess returns in US dollars. They do not consider the additional return that may be earned by investing the capital in money markets.

Portfolios are constructed using the optimization problem described in Proposition 4 with equal risk budgets. Any remarks about equal weights and equal risk contributions also apply to more general budget-weighting and risk-budgeting portfolios.

Calculations are run using Python 3.7.6. Numerical optimization relies on the SLSQP algorithm as implemented in the SciPy library. The variance of the portfolio is the optimization criterion. Its Jacobian is used in the algorithm. The maximum number of iterations is $10^{5}$ and the algorithm is stopped using a precision of $10^{-16}$.

If −1<α<1, the negative alpha-log takes a finite value at 0 with an infinite slope. This combination may lead to numerical instabilities when using gradient methods. When α is close to −1, the derivative exerts a repulsion force as soon as any weight decreases too much. When α is close to 1, the constraint becomes almost linear. Numerical instabilities arise in the middle, when alpha is close to 0. Portfolio weights are constrained to be higher than $\epsilon =10^{-5}$ in order to avoid numerical instability.

This study relies on weekly returns. For some figures and tables that illustrate portfolio weights, covariances are estimated over a period of time that is specified in the title or footnotes. For simulating risk and returns, portfolios are rebalanced every four weeks with a lag of one week. A first portfolio is selected on Wednesday 1 January 1997 and traded one week later at the close. Volatility is the risk measure, and sample covariance matrices are estimated on data from the previous 156 weeks—approximately 3 years. Portfolios are simulated iteratively with no look-ahead bias.

The risk and return of a simulated portfolio are measured using data from the start of the simulation in January 1997 to the end, in August 2022. The Sharpe ratio is the ratio of excess returns to annualized volatility. CVaR is the conditional value-at-risk for a quantile of 1% [2]. Max drawdown is the maximum observed loss from peak to trough as a percentage of peak value [44]. Turnover is the average of absolute rebalancing as a percentage of the notional of the portfolio and over all monthly trading dates.

The diversity of a portfolio is measured using the negative Shannon entropy of weights. Duplication invariance, or lack of it, is identified using the following indicator:

**Definition** **2.** *For any portfolio construction process that leads to a portfolio y and for any asset i in a universe of n assets, the individual sensitivity to duplication is defined by duplicating asset i in the universe and finding an optimal portfolio in the universe of n+1 assets. The weights respective to the asset and its duplicate are then added. This new portfolio of n assets is noted $y^{i}$. The individual sensitivity to duplication is the Hellinger distance $h\left(y,y^{i}\right)$ (see Equation (A14)) between the two portfolios.*

*The duplication sensitivity of the portfolio is the average of these individual sensitivities:*

(30) $$\Delta =\frac{1}{n}\sum_{i=1}^{n}h\left(y,y^{i}\right)$$

In the case of alpha risk parity with α<1, the optimization problems with duplication have a unique solution because the constraint on $F_{\alpha }$ is strictly convex. In the case of minimum-risk portfolios, the solution is not unique. However, all solutions lead to the same allocations $x^{i}$ once exposures to duplicated assets are merged.

Covariance shrinkage—when applied—refers to measuring covariance using the following formula: $\Sigma \left(k\right)=k\;I_{n}+\left(1-k\right)\;\Sigma$. Σ is the matrix of covariances measured over a given time window. $I_{n}$ is the identity matrix with as many entries as assets in the investment universe. An optimal estimator for *k* is proposed in [45]. Following [5], covariance is shrunk towards the identity matrix by increasing *k* from 0 to 1.

## 4. Results

### 4.1. Allocation within the Reference Universe

#### 4.1.1. Diversity and Duplication Sensitivity

**Figure 2:**
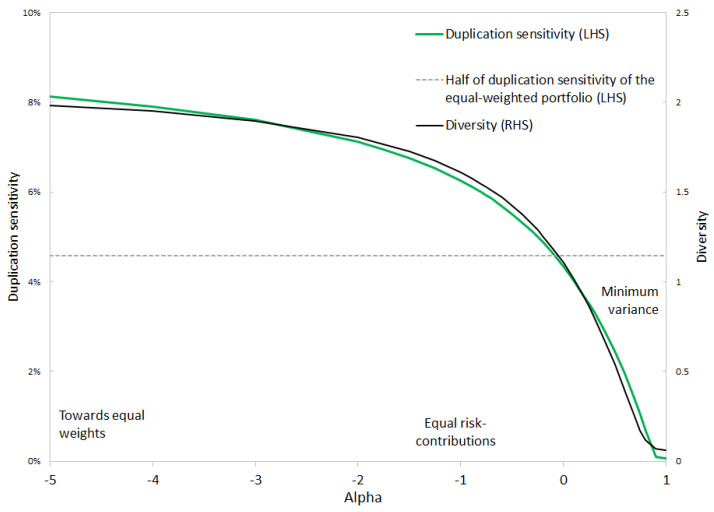
Duplication sensitivity and diversity. Simulation based on data from 7 August 2019 to 10 August 2022. Source: BNP Paribas, Bloomberg.

Following [4], Figure 2 confirms that the equal risk contributions portfolio is not invariant to duplication. While minimum variance is invariant to duplication, the logarithmic constraint that plays a role in equal risk contributions is responsible for a higher sensitivity to duplication.

By increasing α, duplication sensitivity is gradually decreased. The portfolio becomes more concentrated on the assets that offer the lowest risk and the best diversification potential. The diversity of the allocation also decreases.

In terms of diversity and duplication sensitivity, the equal risk contributions portfolio is quite close to the equal-weighted portfolio. The risk minimization algorithm is tightly constrained by the log-barrier. Increasing α loosens the constraint. In Figure 2, duplication sensitivity is reduced by half when α increases from −∞—for equal weights—to α=−0.1. At this level, the alpha risk parity portfolio is a balanced compromise between equal weights and minimum risk.

#### 4.1.2. Risk and Returns

Table 2 shows the risk metrics, Sharpe ratio, and turnover of portfolios for various values of α.

- Volatility decreases continuously with α. The minimum-variance portfolio has a minimum out-of-sample volatility.
- After normalization by volatility, the maximum drawdown is worst for equal weights and fairly stable across the range of α considered here.
- Tail risk—as measured by CVaR—is a higher multiple of volatility when α becomes too close to 1. There is some risk in extreme portfolio concentration.
- Equal weights and minimum variance offer suboptimal Sharpe ratios.
- The equal-weighted portfolio does not have the lowest turnover. Turnover is minimal for equal risk contributions and increases gradually when alpha risk parity becomes close to minimum variance.

Diversity does not mean diversification. Equalizing risk contributions is not optimal in terms of risk because the equal-weighted portfolio is suboptimal. At the other end of the scale, the higher concentration of the minimum-variance portfolio may not be acceptable for most asset managers and may expose investors to excessive tail risk.

Using alpha risk parity with an intermediary level of α may be a good compromise. In Table 2, using α=−0.2 optimizes the Sharpe ratio and offers an almost-minimal ratio of CVaR to volatility. This level of α is close to the point at which duplication sensitivity is halved in Figure 2.

**Figure 3:**
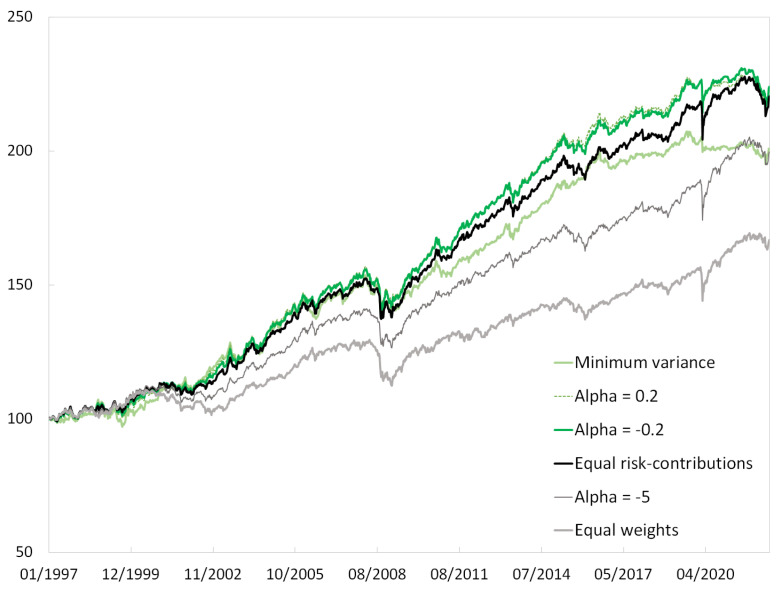
Alpha risk parity portfolios. The portfolios are rescaled in such a way that their volatility equals that of the equal risk contributions portfolio. Source: BNP Paribas, Bloomberg.

The returns of selected alpha risk parity portfolios are depicted in Figure 3.

#### 4.1.3. Alpha Risk Parity and Shrinkage

**Figure 4:**
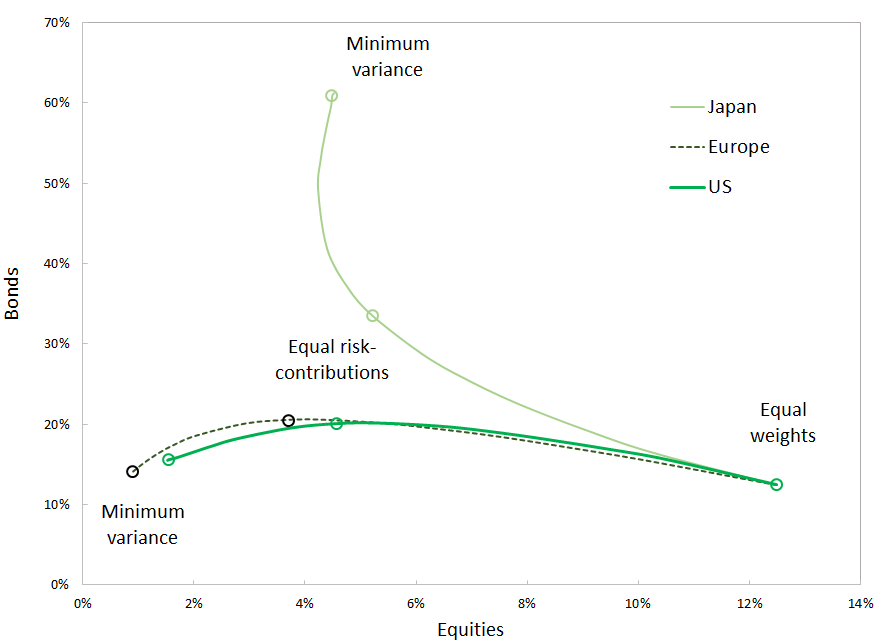
Allocation into equities and bonds for the three regions represented in the investment universe. Allocation based on risk metrics measured over 1994–2022. Source: BNP Paribas, Bloomberg.

As shown in Figure 4, the alpha log-barrier forces the portfolio away from minimum variance and towards equal weights.

**Figure 5:**
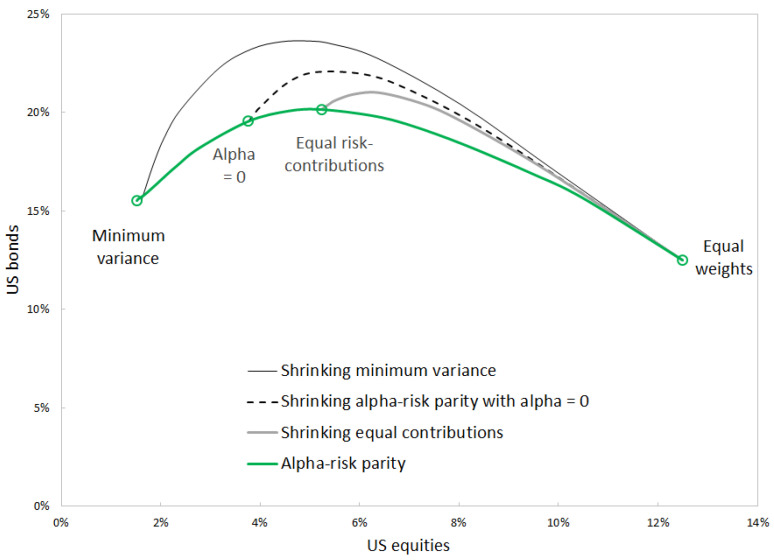
Allocation into US equities and bonds using alpha risk parity and applying covariance shrinkage to selected portfolios. 1994–2022. Source: BNP Paribas, Bloomberg.

Covariance shrinkage is another way to bring the minimum-variance portfolio closer to equal weights. Figure 5 compares the two approaches—using alpha risk parity with decreasing α or using covariance shrinkage.

The two approaches can be used in order to bring the minimum-variance portfolio closer to equal weights; however, they are not strictly equivalent. Equal risk contribution portfolios cannot be obtained by applying covariance shrinkage to the minimum-variance portfolio. Each alpha risk parity portfolio determines its own path of covariance shrinkage, along which the optimal-shrinkage portfolio is located. In contrast, alpha risk parity determines a single path that connects minimum variance to equal risk contributions and equal weights.

### 4.2. Allocation Outside the Reference Universe

Table 3 shows what happens when risk budgets are set to 0% for government bonds. Risk budgets are evenly split across all other indices.

Bonds are selected because they were negatively correlated with other assets on average over the period. When α=1, risk is minimized with a minimum exposure to equities and commodities. The portfolio is concentrated with a large exposure to gold. Decreasing α brings assets with a positive risk budget closer to equal weights. This allocation is not optimal for risk. In order to mitigate risk, the portfolio is reallocated towards bonds—although no risk budget is allocated to this asset class.

## 5. Discussion

Alpha risk parity leads to a continuum of portfolio optimization problems that includes risk budgeting (or equal risk contributions in the particular case of equal reference weights), minimum risk, and converges to the budget-weighted portfolio as a limit case. This observation confirms and complements earlier findings on risk budgeting being a compromise between the two other approaches [3,4]. Within the framework of alpha risk parity, all portfolios from the continuum verify a closed-form risk-budgeting equation. Moreover, risk budgeting is associated with a well-identified parameter—α=−1.

Another continuum was identified in [5]. That continuum results from covariance shrinkage and does not connect the three portfolios. Any alpha risk parity portfolio can be brought closer to a reference portfolio by using covariance shrinkage or by decreasing the value of α. While optimal levels of covariance shrinkage have been proposed [45], a theory for determining the optimal level of α remains to be built. However, optimal covariance shrinkage relies on a number of assumptions that are not always verified in practice. Determining how to exactly determine optimal shrinkage has been subject to debate [46].

Within the framework of alpha risk parity, optimal alpha can be determined geometrically—for example, by starting with the minimum-variance portfolio and looking for the value of α that reduces entropy by half. Alternatively, duplication sensitivity may be used as a criterion.

From this standpoint, it is interesting to note that the risk-budgeting portfolio is not an entirely balanced compromise between minimum risk and the budget-weighted portfolio. If the latter portfolio is not optimal in terms of risk and returns—and the odds are that it is not—then the risk-budgeting portfolio may not be optimal either. Within the framework of alpha risk parity, increasing α is a simple means of decreasing aversion to small weights and giving a bigger role to risk optimization. At the other end of the scale, unfettered risk minimization leads to excessive concentration and high tail risk. As mentioned in [9], the available statistical information is not likely to be perfect. Therefore, some form of compromise is needed.

Following [4], another alternative may be to apply the risk-budgeting algorithm iteratively. It may be interesting to compare this approach with that of alpha risk parity. Lack of granularity in the iterative allocation may make it difficult to identify the right level of α. Moreover, iteration comes at a cost in terms of numerical complexity. In contrast, alpha risk parity is easily solved using the same numerical techniques that are involved in risk budgeting.

## 6. Conclusions

Alpha risk parity applies techniques from information theory and geometry to portfolio allocation. Using Tsalli’s *q*-logarithm and *q*-divergence leads to optimization problems that can be described in simple terms (see Proposition 4) and that rely on solid theoretical foundations and extend the risk-budgeting variational problem. Using the α-parametrization ensures a form of self-duality and compatibility with Amari’s *f*-divergences, which can be used for more general allocation problems. The risk contribution of all assets is given by a closed-form formula. By adjusting α, portfolio managers can let risk contributions deviate from risk budgets and strengthen or weaken the role of risk budgets as a reference portfolio.

## Figures and Tables

**Table 1:** A multi-asset reference universe.

Market	Index	Bloomberg Ticker
US equities	BNP Paribas US Equity Futures	BNPIFUS
EU equities	BNP Paribas Eurozone Equity Futures	BNPIFEU
Japan equities	BNP Paribas Japan Equity Futures	BNPIFJP
10y Bonds US	BNP Paribas Bond Futures US Tsy 10Y	BNPIFU10
10y Bonds DE	BNP Paribas Bond Futures Germany 10Y	BPBFE10
10y Bonds JP	BNP Paribas Bond Futures Japan JGB 10Y	BPBFJ10
Gold	S&P GSCI Gold Index CME	SPGSGCP
Commodities	Bloomberg Commodity ex-Agriculture and Livestock Capped	BBUXALC

Source: BNP Paribas, Bloomberg.

**Table 2:** Risk and returns of alpha risk parity portfolios.

Alpha	−∞	−5	−1	−0.5	−0.2	0	0.2	0.5	1
**Portfolio**	Equal weights		ERC			Hellinger			Min variance
**Volatility**	8.1%	5.1%	3.7%	3.4%	3.2%	3.1%	3.0%	2.8%	2.7%
**Sharpe**	0.532	0.742	0.853	0.867	0.871	0.870	0.864	0.843	0.754
**MDD/vol**	−3.37	−2.78	−2.70	−2.74	−2.79	−2.83	−2.87	−2.92	−2.80
**CVaR/vol**	4.02	4.07	4.00	3.95	3.96	3.97	4.01	4.06	4.14
**TurnOver**	2.7%	2.9%	2.6%	2.7%	2.7%	2.8%	2.9%	3.2%	4.0%

1997–2022. Source: BNP Paribas, Bloomberg.

**Table 3:** Allocation with a risk budget of 0% for bonds.

Alpha	−3000	−5	−1	−0.5	−0.2	0	0.2	0.5	1
**US bonds**	16.5%	15.3%	13.4%	13.0%	12.5%	12.1%	11.4%	10.5%	5.0%
**DE Bonds**	12.7%	12.6%	12.7%	12.3%	12.3%	12.1%	12.1%	11.4%	10.4%
**JP bonds**	13.8%	13.2%	12.6%	12.5%	12.2%	12.2%	11.9%	11.9%	11.9%
**US eq.**	11.4%	11.7%	12.3%	12.7%	13.0%	13.3%	13.8%	15.0%	24.9%
**EU eq.**	11.4%	10.8%	9.8%	9.4%	9.0%	8.7%	8.3%	7.2%	0.0%
**JP eq.**	11.4%	11.2%	10.9%	10.8%	10.7%	10.6%	10.6%	10.5%	10.6%
**Gold**	11.4%	13.4%	16.2%	17.3%	18.2%	19.0%	20.0%	22.0%	29.5%
**Commodities**	11.4%	11.8%	12.1%	12.1%	12.1%	12.0%	11.9%	11.4%	7.7%

Allocation based on risk metrics measured over 1994–2022. Source: BNP Paribas, Bloomberg.

## Data Availability

Not applicable.

## Abbreviations

The following abbreviations are used in this manuscript:

RC	Risk contribution
KL	Kullback–Leibler
MDD	Maximum drawdown
CVaR	Conditional value at risk

## Appendix A. Minimum-Risk Portfolios

Long-only minimum-risk portfolios are a solution to the following problem:

(A1) $$\min\left\{R\left(x\right)|x\geq 0,\;\sum_{i=1}^{n}x_{i}\geq 1\right\}.$$

Due to the constraint, the optimal portfolio *x* cannot be 0. The Lagrangian of this problem is $L\left(y,\mu ,\lambda \right)=R\left(y\right)-\mu \sum_{i}x_{i}-\sum_{i}\lambda_{i}x_{i}$. Differentiating leads to $\partial_{i}R=\mu +\lambda_{i}$ for all *i*. Multiplying by $x_{i}$ leads to $RC_{i}=\mu \;x_{i}+\lambda_{i}\;x_{i}$. However, $\lambda_{i}\;x_{i}=0$ due to complementary slackness. Therefore, $RC_{i}=\mu \;x_{i}$. Summing over all assets shows that μ=R(x)>0. Equation (3) follows. The long-short minimum-risk portfolio verifies the same risk-budgeting equation.

As constraints are linear and the risk criterion may not be strictly convex, there may be multiple solutions to the minimum-risk problem (A1). If the risk criterion is strictly convex on sets of constant exposure, there is only one minimum-risk portfolio.

## Appendix B. The q-Logarithm and Related Functions

- The q-logarithm satisfies the following quasimorphism property.
  (A2) $$\mathrm{For}\;\mathrm{all}\;\lambda ,x>0,\;\log_{q}\left(\lambda x\right)=\lambda^{1-q}\log_{q}\left(x\right)+\log_{\alpha }\left(\lambda \right).$$
- The inverse of the q-logarithm is the q-exponential. For q=1 and α=−1, this inverse is the classical exponential function. For other values of the entropic index and using the alpha parametrization,
  (A3) $$\exp_{q}\left(y\right)={\left(1+\frac{1+\alpha }{2}y\right)}^{\frac{2}{1+\alpha }}.$$
  Of course, $\exp_{q}\left(0\right)=1$. Regarding the domain of definition, if α<−1, $\exp_{q}$ is defined for any $y<-\frac{2}{1+\alpha }$. This upper bound is positive. If −1≤α, $\exp_{q}$ is defined for any y. For any nonnegative number y from the domain of definition, $\exp_{q}\left(y\right)\geq 1$.
- The quasimorphism of the q-logarithm implies a similar property for the $F_{\alpha }$ log-barrier defined in Equation (11).
  (A4) $$\mathrm{For}\;\mathrm{all}\;\lambda >0,\;\mathrm{for}\;\mathrm{all}\;y\in R_{+}^{n},\;F_{\alpha }\left(b,\lambda y\right)=\lambda^{1-q}F_{\alpha }\left(b,y\right)-\log_{q}\left(\lambda \right).$$

## Appendix C. Using the Negative q-Logarithm as a Barrier

If α≤−1, $-\log_{q}\left(0\right)=+\infty$. If −1<α<1, the negative q-logarithm takes a finite value at 0. However, its derivative converges to −∞, and its subgradient is empty. For any function f and nonnegative multiplier μ, $f-\mu \;\log_{q}$ cannot be minimal at 0 because its subgradient is empty [47]. Therefore, alpha risk parity portfolios have positive weights if α<1.

## Appendix D. Simple Formulas for Alpha Risk parity

- In the case of volatility-based equal risk contribution portfolios with constant correlation, Lagrangian (5) can be symmetrized by substracting $\mu \sum_{i}b_{i}\log\left(\sigma_{i}\right)$ and using the morphism property of the logarithm. Considering squared volatility, the problem is solved by minimizing $F(\sigma_{1}y_{1},\ldots ,\sigma_{n}y_{n})$ with:
  (A5) $$F\left(z_{1},\ldots ,z_{n}\right)=\sum_{i}z_{i}^{2}+\rho \;\sum_{i\neq j}z_{i}z_{j}-\mu \sum_{i}b_{i}\log\left(z_{i}\right).$$
  A symmetric and convex function achieves its minimum on the diagonal. Therefore, $\sigma_{i}{\tilde{y}}_{i}$ is constant across assets.
- If α≠−1, volatility-based alpha risk parity portfolios with uncorrelated assets are determined by minimizing the following Lagrangian criterion:
  (A6) $$L\left(y,\mu \right)=\sum_{i}y_{i}^{2}\sigma_{i}^{2}-\mu \;\frac{2}{1+\alpha }\sum_{i}b_{i}^{\frac{1-\alpha }{2}}y_{i}^{\frac{1+\alpha }{2}}.$$
  up to an additive constant. Taking the derivative according to $y_{i}$ directly leads to Equation (12).
- If the correlation is constant and different from 0, Lagrangian (A6) is symmetric if volatility is constant across assets. In this case, the equal-weighted portfolio solves the alpha risk parity problem. If volatility varies across assets and α≠−1, Lagrangian (A6) is not symmetric. The portfolio must be determined using numerical optimization.

## Appendix E. Independence from the Constraints

In this section, α≤1 in line with Definition 1 for alpha risk parity portfolios. q is the associated entropic index.

**Proposition** **A1.**
*For any two constraints $c_{1}$ and $c_{2}$, there is a one-to-one mapping between admissible sets $S_{q}\left(c_{1}\right)\to S_{q}\left(c_{2}\right),\;y\mapsto \lambda \;y$. λ is determined by:*

(A7)
$$\lambda =\frac{\exp_{q}\left(-c_{2}\right)}{\exp_{q}\left(-c_{1}\right)}.$$

**Proof** **of** **Proposition A1.** λ is determined by substituting $c_{1}$ for $F_{\alpha }\left(b,y\right)$ and $c_{2}$ for $F_{\alpha }\left(b,\lambda y\right)$ in quasimorphism Equation (A4):

(A8) $$c_{2}=\lambda^{1-q}c_{1}-\log_{q}\left(\lambda \right).$$

Equation (A7) follows. Therefore, $y\in S_{q}\left(c_{1}\right)\Leftrightarrow \lambda \;y\in S_{q}\left(c_{2}\right)$. □

**Proof** **of** **Proposition 2.** According to Proposition A1 applied to $c_{1}=0$ and $c_{2}=c$, there is a one-to-one mapping $S_{q}\left(0\right)\to S_{q}\left(c\right),\;y\mapsto \exp_{q}\left(-c\right)\;y$. This application maps $y_{q}^{*}\left(0\right)$ into $y_{q}^{*}\left(c\right)$. □

## Appendix F. The Risk-Budgeting Equation

**Proof** **of** **Proposition 6.** The Lagrangian associated with problem (10) is:

(A9) $$L\left(y,\mu \right)=R\left(y\right)-\mu \;\sum_{i}b_{i}\;\log_{q}\left(\frac{y_{i}}{b_{i}}\right)$$

The first-order condition is:

(A10) $$\mathrm{for}\;\mathrm{all}\;i,\;\partial_{i}R=\mu \;{\left(\frac{y_{i}}{b_{i}}\right)}^{\frac{\alpha -1}{2}}.$$

Multiplying by $y_{i}$ implies that ${\mathrm{RC}}_{i}=\mu \;b_{i}\;{\left(\frac{y_{i}}{b_{i}}\right)}^{\frac{1+\alpha }{2}}$ for each asset i. Summing over assets, it can be checked that μ>0 and that $\frac{{\mathrm{RC}}_{i}}{R}\left(y\right)\;\propto \;b_{i}\;{\left(\frac{y_{i}}{b_{i}}\right)}^{\frac{1+\alpha }{2}}$. Thanks to the homogeneity of R, both sides of this equations can be rescaled in such a way that normalized portfolio $\tilde{y}$ is substituted for y. Risk-budgeting Equation (19) follows. □

## Appendix G. Why Alpha Risk Parity Is a Maximum-Utility Problem

**Proof** **of** **Proposition 7.** The solution $y_{q}^{*}$ of problem (10) minimizes risk on all long-only portfolios, including those with constrained exposure $\sum_{i}y_{i}=\lambda^{*}=\sum_{i}{\left(y_{q}^{*}\right)}_{i}$. Therefore, $y_{q}^{*}$ is solution to $\min\left\{R\left(y\right)|y\geq 0,\;\sum_{i}y_{i}=\lambda^{*},\;\;F_{\alpha }\left(b,y\right)\leq c\right\}$. It is interesting to note that $\sum_{i}y_{i}=\lambda^{*}\Leftrightarrow \sum_{i}x_{i}=1$, where $x=y/\lambda^{*}$. Applying Proposition A1 to $c_{1}=c^{*}$, $c_{2}=0$ and $\lambda =\lambda^{*}$ exhibits a one-to-one mapping $S(c^{*})$$\to S\left(0\right),\;y\mapsto \lambda^{*}\;y$. The constraint $c^{*}$ of the rescaled problem is directly determined by Equation (A8):

(A11) $$c^{*}=\lambda^{*\;-(1-q)}\;\log_{q}\left(\lambda^{*}\right)=-\log_{q}\left(\frac{1}{\lambda^{*}}\right).$$

Alpha risk parity portfolio $\tilde{y}$ is a solution to the divergence-constrained problem:

(A12) $$\min\left\{R\left(x\right)|x\geq 0,\;\sum_{i}x_{i}=1,\;-\sum_{b_{i}>0}b_{i}\log_{q}\left(\frac{x_{i}}{b_{i}}\right)\leq c^{*}\right\}.$$

If $c^{*}$ were negative, the admissible set of that problem would be empty. However, this is not the case. Nonnegativity of $c^{*}$ can also be checked directly. As noted in Proposition 4, $\lambda^{*}\geq 1$. Equation (A11) implies that $c^{*}\geq 0$. If α>−1, (A11) implies that $c^{*}<\frac{2}{1+\alpha }$ verifies condition (9). □

## Appendix H. Using Divergence Functions

Divergence functions [42,48] associate a positive number with any pair of distinct points on the simplex or on more general manifolds. Divergences that are defined on the simplex are also called statistical divergences.

Divergences are required to be strictly convex locally. They may be interpreted as squared distances. However, they are not required to be symmetric. In the context of portfolio allocation, symmetry is not needed. The flipside of using nonsymmetric functions is that careful attention must be paid to the order of the arguments. The dual of a divergence is defined by inverting the order of the arguments: $D^{*}\left(b\|x\right)=D\left(x\|b\right)$.

Divergences are decomposable when they are expressed as sums over individual coordinates. Invariance is another useful property. It ensures that aggregating assets into buckets does not increase the divergence between two portfolios. It can be shown that the invariant and decomposable divergences with at least two assets are the f-divergences. The f-divergences [49,50] are defined for all x in the simplex as:

(A13) $$D_{f}\left(b\|x\right)=\sum_{b_{i}>0}b_{i}f\left(\frac{x_{i}}{b_{i}}\right).$$

Function f is differentiable, convex and verifies f(1)=0. Once squared, the Euclidean distance is a divergence. However, it is not invariant. This is one reason why f-divergences may be a better choice for portfolio optimization than classical sums of squares.

Bregman divergences [51] are a class of divergences that are considered as simple. They can be defined on the simplex, positive measures or more general sets. They rely on a convex function and induce a double—or dual—linear parametrization that can be useful in computations. Amari describes these divergences as flat [42].

The KL divergence and its dual stand out as the only flat f-divergences that are defined on the open simplex when n≥2 [52]. Its dual is closely related to Shannon’s entropy of portfolio x. Amari’s alpha divergences are the only flat f-divergences of positive measures—also when n≥2 [8]. They are not flat on the open simplex.

When α=0, the square of the f-divergence is a multiple of the Hellinger distance h that is defined for any b and x by

(A14) $$h^{2}\left(b,x\right)=\frac{1}{2}\;\sum_{i}{\left(\sqrt{b_{i}}-\sqrt{x_{i}}\right)}^{2}.$$
